# Effects of Electromyographic Biofeedback-Assisted Exercise on Functional Recovery and Quality of Life in Patients after Total Hip Arthroplasty: A Randomized Controlled Trial

**DOI:** 10.3390/jpm13121716

**Published:** 2023-12-15

**Authors:** Tomislav Kokic, Roman Pavic, Matko Vuksanic, Stjepan Jelica, Antun Sumanovac, Tihomir Banic, Helena Ostović, Iva Sklempe Kokic

**Affiliations:** 1 Faculty of Medicine, Josip Juraj Strossmayer University of Osijek, 31000 Osijek, Croatia; 2 Orthopaedics and Trauma Department, General County Hospital Vinkovci, 32100 Vinkovci, Croatia; 3 Faculty of Kinesiology Osijek, Josip Juraj Strossmayer University of Osijek, 31000 Osijek, Croatia; 4 Clinical Hospital of Traumatology, University Hospital Centre “Sestre Milosrdnice”, 10000 Zagreb, Croatia; 5 Bizovacke Toplice Rehabilitation Hospital, 31222 Bizovac, Croatia; 6 Department of Health Studies, College of Applied Sciences “Lavoslav Ruzicka” in Vukovar, 32000 Vukovar, Croatia; 7 Faculty of Medicine, University of Zagreb, 10000 Zagreb, Croatia; 8 Department of Surgery, University Hospital Centre Zagreb, 10000 Zagreb, Croatia; 9 Clinical Department of Anesthesiology, Reanimatology and Intensive Care Medicine, University Hospital Dubrava, 10000 Zagreb, Croatia

**Keywords:** biofeedback, hip arthroplasty, quality of life, physiotherapy, motor function, therapeutic exercise, recovery

## Abstract

The goal of the trial was to examine the effects of adding electromyographic biofeedback (EMG-BF) to the conventional program of physiotherapy after total hip arthroplasty (THA) on functional recovery and quality of life. The trial was designed as a prospective, interventional, single-blinded randomized controlled study. Ninety patients were randomized into an experimental group (EG) (*n* = 45; mean age 63.9 ± 8.8) and control group (CG) (*n* = 45; mean age 63.9 ± 9). All patients received 21 days of physiotherapy which consisted of therapeutic exercise (land-based and aquatic), electrotherapy, and education. Electromyographic biofeedback was added to a portion of the land-based exercise in EG. The Hip Disability and Osteoarthritis Outcome Score (HOOS), Numeric Rating Scale (NRS), Short Form Health Survey-36 (SF-36), use of a walking aid, 30 s chair stand test (CST) as well as the Timed Up and Go (TUG) test were used for outcome measurement. A higher proportion of the participants in both groups did not need a walking aid after the intervention (*p* < 0.05). All participants improved their 30 s CST and TUG results (*p* < 0.001), as well as their NRS and HOOS scores (*p* < 0.05). No significant differences between the groups were found. There were no additional benefits from adding EMG-BF to the conventional physiotherapy protocol.

## 1. Introduction

Osteoarthritis (OA) of the hip is a very common musculoskeletal degenerative disease in elderly people and presents one of the leading causes of global disability [1]. In advanced stages, patients become disabled, suffer from pain, and their quality of life is decreased. Total hip arthroplasty (THA) is an effective and final solution for patients with advanced hip OA. It is one of the most frequent orthopedic surgical procedures, and considered as the “surgery of the century” [2,3]. Hip replacement surgery has been revolutionized in the last decades due to the development of new materials, and improvements in design and surgical technique [2,4]. It has a low revision rate, excellent outcomes, and low mortality [5,6].

Although THA is very successful and the final result is pain reduction, improvement of quality of life, and functional recovery in the majority of patients, around 7% of patients are not satisfied with the surgery outcomes [7]. However, the level of satisfaction with the surgery outcomes in the study conducted by Anakwe et al. [7] was associated with self-reported mental health, a history of depression, or the presence of significant arthritis of another major joint. Halawi et al. [8] also reported that 11% of patients were not satisfied with the outcomes of THA, mostly due to persistent pain and functional limitations. Efficient postoperative rehabilitation is crucial for the optimal outcomes [9,10]. After the surgery, patients suffer from pain and their muscular strength and neuromuscular control is impaired, affecting function and satisfaction [11,12]. Therapeutic exercise for improvement of muscle strength, endurance, and neuromuscular control facilitates functional recovery after THA [9].

Lower extremity muscle mass is one of the key indicators of physical ability in the older population with mobility limitation, and muscle strength is a strong independent predictor of functional impairment [13]. It has been suggested that there is an association between m. quadriceps femoris muscle strength and functional outcomes, e.g., ability to perform activities of daily life, walking, standing up, or using the stairs, in patients after THA [14]. Likewise, there is strong evidence regarding the reduced muscle mass of m. quadriceps femoris in patients with hip OA, and the deterioration of m. quadriceps femoris function after THA [15,16].

Electromyographic biofeedback (EMG-BF) provides real-time information regarding the level of muscular activity during exercise, which would otherwise be unknown [17]. While there is no evidence regarding the beneficial effects of EMG-BF after THA, there are reports in the literature on the potential of this method for pain reduction, improvements in m. quadriceps femoris strength and range of motion, as well as improvement in the overall function in patients after different orthopedic surgical procedures [18,19,20,21]. Wang et al. [18], in their systematic review with meta-analysis, reported on the use of technology-based rehabilitation interventions after THA and total knee replacement (TKA). Technology-based interventions, in comparison to the conventional interventions, were more effective in pain reduction and functional improvements for TKA patients. However, there is still a research gap regarding the use of technology-based interventions in THA patients as there is only very limited and low-quality evidence regarding the benefit. Pfeufer et al. [20] and Xie et al. [21] reported on the effectiveness of biofeedback for improving gait symmetry, pain reduction, and increasing the activity level in TKA patients, but it is questionable whether the effects could be translated to THA patients.

The goal of this trial was to investigate the effects of EMG-BF added to the standard program of physiotherapy, which consisted of land-based and aquatic exercise, electrotherapy, and education in patients after THA on functional recovery and quality of life. We hypothesized that EMG-BF supplementation would improve motor function and health-related quality of life.

## 2. Materials and Methods

### 2.1. Study Design

A single-blinded, prospective randomized controlled trial was performed with two parallel groups of participants. The trial was not blinded for participants, but the assessors were blinded. The participants were randomized by block randomization, using a computerized service, into two groups: experimental (EG) and control (CG). The Ethics Committee of the Bizovacke Toplice Rehabilitation Hospital, Bizovac, Croatia, and the Ethics Committee of the Faculty of Medicine, Josip Juraj Strossmayer University of Osijek, Osijek, Croatia approved the trial. It was conducted according to the Declaration of Helsinki. The study was prospectively registered with The Australian New Zealand Clinical Trials Registry (ACTRN12622001130752).

### 2.2. Participants

The participants in this trial were 90 patients after THA admitted to inpatient postoperative rehabilitation and randomized to the EG or CG. Recruitment of the participants was on their first day of hospitalization, by direct contact. All of the patients admitted to inpatient postoperative rehabilitation in the facility were informed about the research and asked to participate if they fulfilled the inclusion criteria. We included participants of both genders aged between 18 and 79 years who had independent walking capability before the surgery. They had to be fluent in the Croatian language. Participants who underwent a revision of the THA, those not able to participate in the standard hospital physiotherapy program, and those affected by other comorbidities which afflicted their walking capability (e.g., central nervous disorders, severe cardiovascular of respiratory diseases) were excluded from the trial. Patients who were non-ambulatory before THA, where surgery was performed only for pain relief, were also excluded.

The sample size was determined with the G*Power software [22]. The study was powered to detect at least moderate effect sizes. Specifically, we calculated the sample size that would provide us with at least 80% power to detect an effect size of 0.65 using a two-sided significance level of 0.05 and expected dropout rate of 10%. A minimum sample size of 86 participants was required, 43 participants in each group.

### 2.3. Outcome Measures

On the first day of hospitalization an initial interview was performed where general demographic information and the patients’ medical histories were gathered, including body mass, body height, side of the hip replacement, surgical approach, type of prosthesis fixation, name of the hospital, type of institution where the surgery was performed (university or general hospital), and postoperative day. Use of a walking aid was also documented (unilateral crutch, two crutches, walker, or no walking aid). The standard equation was used to calculate each patient’s body mass index (BMI). All the assessments were performed on the first and the last day of the hospital stay. Two self-reported measures of health-related quality of life, the Hip Disability and Osteoarthritis Outcome Score (HOOS), and Short Form Health Survey-36 (SF-36) were used. Pain intensity was measured using the Numeric Rating Scale (NRS). Functional performance was assessed using the Timed Up and Go (TUG) test and 30 s chair stand test (CST). Participants in both groups were divided in two subgroups: those with an earlier start of their rehabilitation and those with a later start of their rehabilitation, and their baseline values were checked for homogeneity between the EG and CG.

The HOOS is a questionnaire developed for evaluating the symptoms and limitations in patients with hip pain with acceptable psychometric properties for patients undergoing THA [23,24,25,26]. It consists of 40 items divided into five subscales: pain (10 items), symptoms (5 items), activities of daily life (17 items), sports/leisure (4 items), and quality of life (4 items). Scoring is performed using a 5-point Likert scale, from 0 to 4. For each subscale, a normalized score was calculated, with values between 0 (extreme symptoms) and 100 (no symptoms).

The Short Form Health Survey-36 (SF-36) is a generic measure of health status not specific for age, disease, or treatment [27]. It consists of 36 items divided into eight health domains [27,28]: physical functioning (10 items), role limitations due to physical health problems (4 items), role limitations due to personal or emotional problems (3 items), vitality (4 items), mental health (5 items), social functioning (2 items), bodily pain (2 items), and general health (5 items). The Croatian version of the questionnaire was used [29]. Each domain is scored on a scale of 0–100, where 0 represents the worst overall health status, and 100 the best health status. The eight domains can be combined into two summary measures: physical component summary (PCS) and mental component summary (MCS) scores. These two distinct summary components were aggregated using the Croatian reference population [30]. The scoring and calculation of the scales were performed by using the survey’s manual [28,31]. SF-36 has acceptable psychometric properties for patients after THA, including adequate responsiveness [32,33,34,35].

The NRS is a reliable and valid method of measuring pain intensity [36,37]. We used an 11-item NRS in which the participant selects a whole number between 0 (no pain) and 10 (worst pain imaginable) that best reflects the intensity of their pain.

The thirty-second CST assesses lower extremity function and lower body strength [38,39]. The subjects are requested to perform as many full stands from a chair in the time period of 30 s. The number of full stands is documented and represents the result of the 30 s CST. The test has excellent test–retest reliability in patients after THA [40].

TUG is a simple and widely used measure of lower extremity function, mobility, and risk of fall [41]. The time required to rise from a chair, walk the distance of 3 m, return back to the starting position, and sit on the chair is measured and represents the result of TUG. It is widely used for outcome assessment after THA, with acceptable psychometric properties, including reliability and predictability of functional abilities [42,43,44].

### 2.4. Intervention

Both groups were included in an inpatient postoperative rehabilitation program for a duration of 21 days. The standard institutional protocol for rehabilitation after THA was used. This consisted of daily sessions of physiotherapy including land-based therapeutic exercise (50 min), aquatic therapy exercise (30 min), application of physical agents (interferential current therapy (ICT) (10 min), electrostimulation (10 min)), and individual education. Each day of rehabilitation followed the same protocol, but therapy was not provided on Sundays. On average, during the 21 days of hospital stay, each patient received 18 days of physiotherapy. All physiotherapy interventions were performed every day. However, education was performed once, at the start of the rehabilitation. The interventions were performed face to face, by the physiotherapist in charge of the specific patient. Physiotherapists monitored adherence to the treatment. All interventions were performed by two experienced physiotherapists. Likewise, all assessments were performed by the team of two experienced physiotherapists, who were not involved in the direct provision of rehabilitation interventions.

The land-based therapeutic exercise program included 20 exercises. There were seven variations of isometric exercises for muscles of the thigh with a focus on m. quadriceps femoris, and thirteen dynamic exercises for lower extremity muscle strength and endurance. Dynamic exercises included a pelvic lift exercise, two variations of an active straight leg raise, abduction of the hip with extended leg from supine position with and without elastic band, hip and leg flexion from a supine position with and without elastic band, hip and leg flexion towards the patient’s chin using the Swiss ball, changing the position of the patient’s leg from the Swiss ball to the therapeutic table in supine position, hip abduction with extended leg from side lying position, hip flexion and extension with extended leg from side lying position, leg flexion and extension from side lying position, and leg extension in sitting position. All exercises were performed for the whole duration of the intervention. The isometric exercises were carried out in sets of one with five repetitions. Every repetition was performed with a maximal effort duration of 5 s. The dynamic exercises were carried out in sets of one or two, and the number of repetitions was 10. The number of sets were decided regarding the participant’s ability. If the patient was unable to perform two sets of every exercise in the protocol, only one set was performed, with the goal of achieving the ability to perform two sets of every exercise in the protocol. An elastic band was used for the progression of dynamic exercises, using bands of different elasticity. During the last 6 days of the intervention ankle weights (1 kg) were added.

An aquatic therapy exercise program was performed by the group. It included 11 exercises for lower body strength and range of motion. Participants performed standing toe raises, standing heel raises, extension of the hip in standing position, flexion of the hip with extended leg while standing in the pool, hip and leg flexion in standing position, hip abduction from standing position, leg extension from standing position, touching ipsilateral hand and knee, hip and leg flexion in supine floating position, hip abduction and adduction in supine floating position, semi-squats, and scissors exercise in supine floating position.

A Myomed 632 device (Enraf-Nonius B. V., Rotterdam, The Netherlands) was used for ICT and electrical stimulation, with the intensity used according to each patient’s tolerance and the manufacturer’s instructions. Individual education was focused on long-term care of the prosthesis, recommended and non-recommended activities, prevention of blood clots, coping with pain, and motor function recovery. Physiotherapists provided the education at the start of the intervention.

Electromyographic biofeedback was added to a portion of the land-based therapeutic exercise in EG using the Myomed 632 device (Enraf-Nonius B. V., Rotterdam, The Netherlands). The threshold for isometric contraction was individually adjusted, according to the manufacturer’s instructions. A superficial electromyography (EMG) unit was used to provide biofeedback. The unit registered electrical activity of the muscle and presented it as visual information during the exercise. The sensitivity of the EMG signal was set to 200 µV to ensure good visibility of the feedback. Both patient and physiotherapist monitored the level of muscle activation with the goal of achieving adequate intensity. The unit was attached to the participants with three electrodes, two EMG electrodes placed on the muscle belly of the m. quadriceps femoris, and one reference electrode placed on the anterior portion of the tibia of the opposite leg. At the beginning of the session, the patient was asked to perform maximal voluntary isometric contraction (MVIC) of the m. quadriceps femoris. This value was recorded in the machine and used to establish the desired threshold, e.g., the minimum intensity of the muscular contraction which should be accomplished during the exercise. Eighty percent of the MVIC was set as the threshold, with the aim being to contract the muscle above the threshold visible on the screen. The goal of the patient during the isometric muscle contractions was to surpass the established threshold which was visible on the screen. The biofeedback-supplemented therapeutic exercise consisted of MVICs of the quadriceps femoris muscle. It was performed for 15 min with contractions lasting 10 s, followed by 10 s periods of relaxation. The control group received the same rehabilitation program, except for the biofeedback-supplemented exercise. Participants from the CG performed this part of the therapeutic isometric exercise without EMG-BF.

### 2.5. Statistical Analyses

Statistical analyses were performed using SPSS 25.0 (IBM, Armonk, NY, USA). The Shapiro–Wilk test was used to determine the normality of the data, and Levene’s test was used to check the homogeneity of the variance. Descriptive statistics were calculated, and are presented as mean and standard deviation (SD) for data with a normal distribution, and median and interquartile range (IQR) for data without a normal distribution. Categorical variables were summarized using frequencies and percentages.

Between-group comparisons were performed using *t*-test for independent samples for data with normal distribution or the Mann–Whitney U test for data without normal distribution. The chi-square test and Fisher’s exact test were used for categorical variables.

Within-group (pre- and post-intervention) comparisons were performed using the *t*-test for paired samples for normally distributed data or Wilcoxon signed-rank test for data without a normal distribution, and chi-square test for categorical variables.

The level of significance was set at *p* < 0.05.

## 3. Results

**Figure 1:**
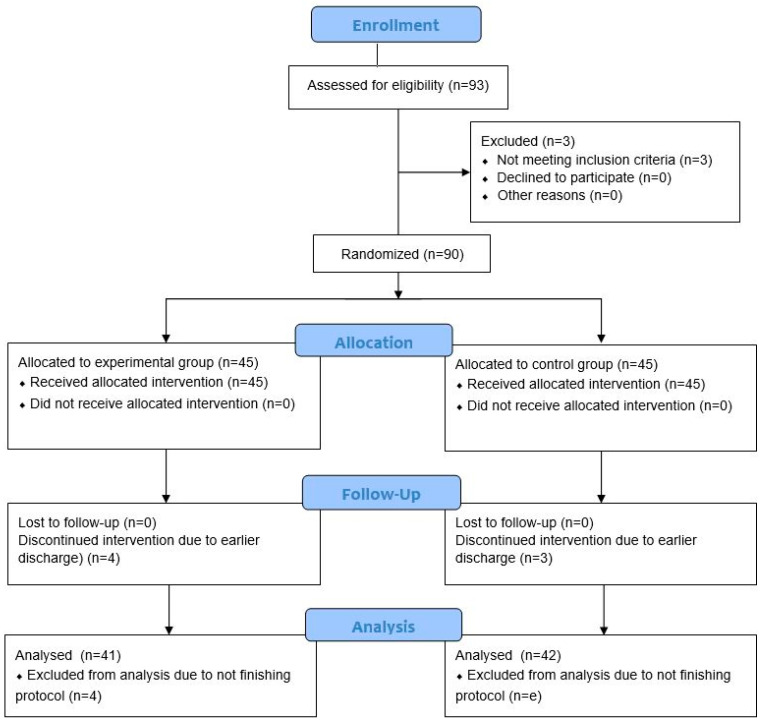
Consolidated Standards of Reporting Trials (CONSORT) flowchart of the study showing recruitment of participants.

A total of 90 participants admitted to inpatient rehabilitation after total hip arthroplasty were finally enrolled in the study. They were randomized to EG and CG groups, 45 patients in each group. Seven participants (7.8%) were lost in the trial, specifically four participants allocated to the EG (8.9%), and three participants allocated to the CG (6.7%) (Figure 1). The final analysis included 83 participants, 41 allocated to the EG, and 42 allocated to the CG. Both groups were well matched, without significant differences (*p* > 0.05) regarding their demographic and anthropometric characteristics, side of the hip replacement, surgical approach, location of the surgery, postoperative day, use of mobility aid, 30 s CST, TUG, NRS, and results of HOOS, and SF-36 questionnaires at the beginning of the trial (Table 1). All patients received cementless prostheses. All participants were hospitalized for 21(0) days receiving the intervention for 18(0) days.

The median of the postoperative day at the start of the trial was 90 (60–120) days. According to that patients in both groups were divided in subgroups: those with an earlier start of their rehabilitation (N = 43), and those with a later start of their rehabilitation (N = 40) (Table 2). The distributions of early and late starters in the EG and CG were without significant differences (*p* = 0.545). In the subgroup of early starters, a significantly higher proportion of patients from the CG were operated on in general hospitals (*p* = 0.022) using the lateral surgical approach (*p* = 0.047). Likewise, in the subgroup of late starters, patients from the EG had slightly better results in the two domains of SF-36: role limitation due to emotional problems (*p* = 0.041) and general health (*p* = 0.044).

### 3.1. Within-Group Analyses

**Figure 2:**
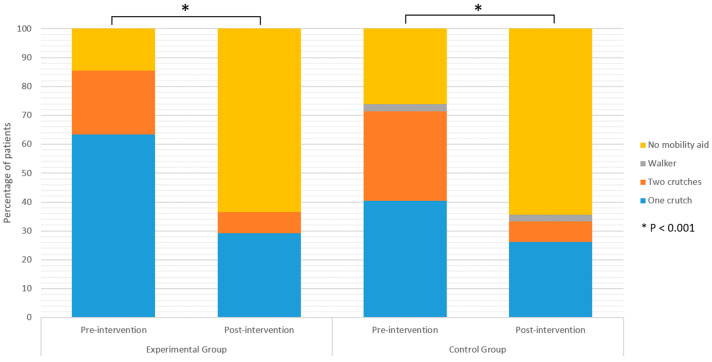
Use of walking aids before and after the intervention.

**Figure 3:**
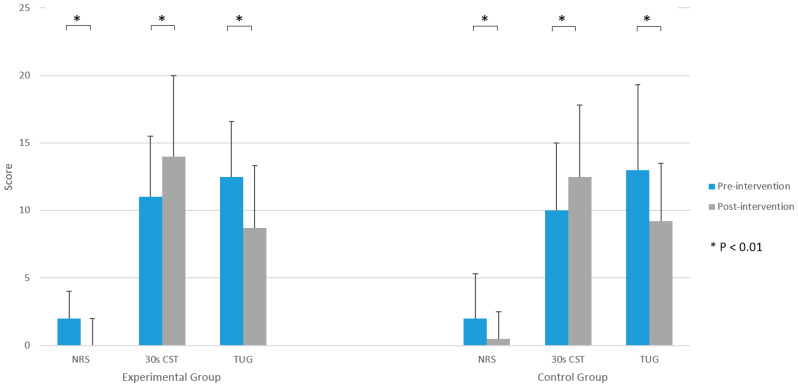
Numeric Rating Scale (NRS), 30 s chair stand test (CST), and Timed Up and Go (TUG) test scores before and after the intervention.

Functional performance from the start till the end of the rehabilitation improved in both groups. A higher proportion of the participants in both groups did not need walking aid after the intervention (*p* < 0.05) (Figure 2). All participants improved their results on the 30 s CST in comparison to the baseline measurement (*p* < 0.001) (Figure 3). In the EG, there was an average improvement of 3.5 stands from a seated position on a chair to standing position during the time frame of 30 s in comparison to the number of stands which participants were able to perform at the beginning of the trial, while in the CG the improvement was 3.1 stands.

The results of the TUG test were also improved in comparison to the baseline measurements for all participants (*p* < 0.001) (Figure 3). In the EG, it was shorter by 3.6 s, and 4.8 s in the CG. The NRS scores also improved for all participants (*p* < 0.001) (Figure 3).

**Figure 4:**
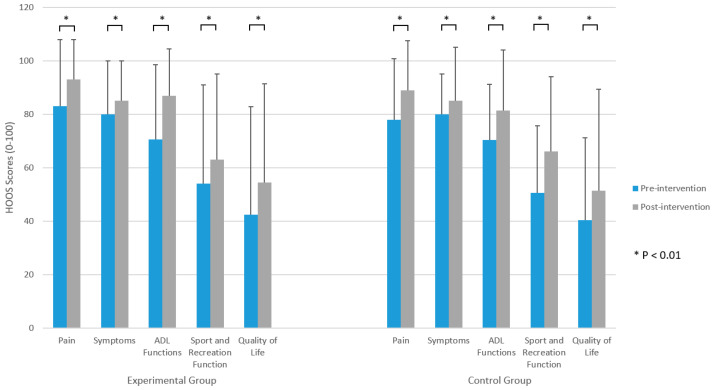
Hip Disability and Osteoarthritis Outcome Score (HOOS) before and after the intervention.

The HOOS scores significantly improved in both groups in all subscales (*p* < 0.05) (Figure 4). The results of the SF-36 before and after the intervention for both groups are shown in Table 3. Participants in the EG significantly improved in all domains of SF-36 except mental health and MCS, while participants in the CG significantly improved in all domains but role physical, role emotional, mental health, and MCS.

### 3.2. Between-Group Analyses

Use of a walking aid, the results of the HOOS and SF-36 questionnaires, NRS, 30 s CST, and TUG results are shown in Table 4. No significant differences between the EG and the CG were found post-intervention regarding the use of a mobility assistive aid (Figure 2). Likewise, the results of the NRS, 30 s CST, and TUG post-intervention did not significantly differ between the groups (Figure 3). The difference between groups in the results of the 30 s CST was borderline (*p* = 0.076), in favour of the EG. However, it was not statistically significant. Furthermore, the results of the HOOS (Figure 4) and SF-36 questionnaire did not differ between the groups. Although the difference in the scores of the role emotional domain of the SF-36 questionnaire (*p* = 0.051) was borderline in favour of the EG, it was above the determined level of significance.

## 4. Discussion

This trial was performed with the aim of investigating the effects of EMG-BF as a supplement to the conventional rehabilitation on functional recovery and quality of life in patients after THA. The results did not confirm our initial hypothesis that the addition of the EMG-BF would yield beneficial effects on pain intensity, quality of life, mobility, and functional performance in comparison to the conventional rehabilitation program consisting of therapeutic land-based and aquatic exercise, electrotherapy, and education. No significant differences between the EG and the CG were determined in use of walking aids, NRS, HOOS, and SF-36 scores at the end of the intervention. Furthermore, there were no significant differences between the groups in the results of the 30 s CST and TUG at the end of the intervention. Although the difference between groups in the scores of the SF-36 domain role emotional and the results of the 30 s CST were borderline, they were above the determined level of significance. To the best of our knowledge, this is the first trial which has examined the effects of EMG-BF-assisted exercise in a THA population.

Biofeedback provides awareness of physiological processes and the possibility to control them [45]. In rehabilitation, it can improve precision during functional tasks, increase patient effort in achieving rehabilitation goals, and reduce the need for ongoing contact with rehabilitation professionals who monitor the implementation of the rehabilitation program [17]. It can be described as a “psycho-physiological mirror” which provides users with a way to monitor physiological signals produced by their body and self-regulate them [46]. Electromyographic biofeedback converts myoelectrical signals to visual or auditory signals, allowing the assessment of the strength of muscular contraction [17]. It is mainly used to improve muscular activity in weak or paretic muscles, to lower the muscle tone in spastic muscles, e.g., for decreasing excessive and inappropriate muscle activation, and for improvement of neuromuscular control [17,45].

Only two previous studies have reported on the use of biofeedback in the rehabilitation of THA patients; however, it was not electromyographic biofeedback, but a limb loading monitoring device [47,48]. White and Lifeso [47] evaluated a treadmill walking program incorporating real-time biofeedback to reduce asymmetric limb loading after THA. The biofeedback helped participants to achieve a more symmetric gait. Isakov [48] evaluated biofeedback in patients referred for full-weight-bearing gait rehabilitation in patients with lower extremity amputation, hip and knee replacement, and after femoral neck fracture. The device significantly improved body weight loading over the affected lower limb.

There were also studies investigating the use of EMG-BF in different orthopedic patients. Draper and Ballard [49] reported on EMG-BF’s superiority in the facilitation of m. quadriceps femoris strength recovery in comparison to electrostimulation after reconstruction of the anterior cruciate ligament (ACL). Norozian et al. [50] compared the effects of usual rehabilitation and the use of adjuvant EMG-BF after ACL reconstruction. After four weeks of the treatment there was a significant increase in m. quadriceps strength and the results of self-reported measures of recovery in the EMG-BF group, but not in the control group.

Likewise, biofeedback was useful in the recovery of patients after meniscectomy [51,52,53]. Akkaya et al. [52] compared outcomes in patients after arthroscopic partial meniscectomy after three different rehabilitation protocols. Those using EMG-BF in addition to home exercise had greater strength of the quadriceps femoris muscle, better results on the Lysholm Knee Scoring Scale, and shorter periods of walking aid use in comparison to patients who were prescribed only a home exercise program or home exercise program and electrical stimulation therapy to the m. quadriceps femoris. Likewise, Kirnap et al. [53] reported better results on the Lysholm Knee Scoring Scale, and improved leg flexion angle and m. quadriceps femoris strength in patients who used EMG-BF.

However, some trials did not confirm the superiority of adding EMG-BF to a conventional rehabilitation protocol in patients with OA and joint replacement surgery. Yilmaz et al. [54] did not find superior effects of EMG-BF in comparison to standard therapeutic exercise in patients with OA of the knee. In our previous study on TKA patients, we also did not find significant additional effects of EMG-BF compared to a conventional rehabilitation program [55]. However, Kondo et al. [56] also performed a trial on patients after TKA and their results led to the conclusion that auditory and visual biofeedback provides better pain relief, which could lead to greater improvements in functional outcomes in postoperative rehabilitation after TKA.

The role of EMG-BF in patellofemoral pain syndrome has also been investigated, with controversial results. While Wise et al. [57] suggested the efficacy of the combination of EMG-BF and strengthening exercise, later studies by Dursun et al. [58] and Yip and Ng [59] did not confirm the superiority of EMG-BF over conventional exercise therapy in patients with patellofemoral pain syndrome. Yip and Ng [59] randomly assigned participants with patellofemoral pain into two groups: exercise only, and exercise supplemented by EMG-BF lasting for 8 weeks. The biofeedback group improved faster than the control group, but without significant differences. Another study performed by Raeissadat et al. [60] investigated the effects of EMG-BF on levels of pain, muscular thickness, and MVIC of the m. vastus medialis in patients with OA of the knee. They did not find significant differences between the control group, which performed isometric exercises, and the experimental group, where isometric exercises were supplemented by EMG-BF, except in the level of pain.

However, Ng et al. [61] reported on the efficacy of the addition of EMG-BF to the exercise program in participants with patellofemoral syndrome. Alonazi et al. [62] reported on the beneficial effects of adding EMG-BF to patellar taping in adult male athletes with patellofemoral pain syndrome. Supplementation of EMG-BF led to significant improvements in pain intensity, functional outcomes, and strength of the m. quadriceps femoris.

Morales-Sánchez et al. [63] reported that EMG-BF is effective in the recovery of m. vastus lateralis in soccer players after a meniscectomy. Krepkovich et al. [64] compared the KneeBright virtual game system and EMG-BF among patients with knee OA and concluded that the novel game system produced better results in knee torque than a conventional EMG-BF system. Still, visual EMG-BF provides a higher MVIC peak torque of the m. quadriceps femoris in comparison to achieving MVIC without biofeedback [65].

Wasielewski et al. [66] reported on the efficacy of EMG-BF for increasing the strength of the quadriceps femoris muscle strength and motor function in patients following ACL reconstruction and meniscectomy, while its use was not equally efficient in chronic disorders like patellofemoral pain syndrome and knee OA. Another recent systematic review reported on the efficacy of EMG-BF in pain reduction, m. quadriceps femoris strength, and overall function in patients after surgical procedures on knee joints [67].

Although the EMG-BF shows potential in the recovery of the m. quadriceps femoris function for patients after surgical procedures on knee joints, the literature on the effects of EMG-BF on patients after THA is nonexistent. While outcomes after THA are undoubtedly improving owing to the development of prosthesis design, a minimally invasive surgical approach, and enhanced recovery protocols, many patients after the surgery have inadequate muscle strength, functional limitations, and pain [12]. This can lead to dissatisfaction and poorer long-term outcomes. Hip and knee muscle strength in patients after hip replacement is impaired for a prolonged period of time [68,69]. Reardon et al. [16] reported on a reduction in ipsilateral m. quadriceps femoris thickness 5 months after THA despite rehabilitation.

Obviously, there is the need to further improve rehabilitation protocols with the goal of avoiding less than optimal functional outcomes and to lower the rate of dissatisfaction after the surgery. Addressing the lower extremity muscle mass and strength could lead to further improvements in patients’ outcomes. While plenty of literature on rehabilitation after THA is available, with numerous published protocols, systematic reviews, and guidelines [70,71,72], specific components and best clinical practice are still not consistent and standardized. The concept of personalized medicine is an emergent and rapidly developing approach to medicine, including rehabilitation, with technology such as EMG-BF supporting it. EMG-BF offers a personalized approach to training and rehabilitation due to its capability to adjust the targets of contraction intensity to individual characteristics and needs. It that way, a more individual approach tailored to specific patient’s needs and expectations is achievable and results in better outcomes for the patient than the traditional “one-size-fits-all” approach.

New devices, rehabilitation regimes, and interventions intended to improve patients’ outcomes after orthopedic surgery are emerging on the market every day. However, their effectiveness and clinical meaningfulness should be investigated to justify their cost and to provide the best standard of care to patients.

While this study confirmed significant improvements after intensive inpatient rehabilitation in both groups of patients after THA, it did not justify the use of EMG-BF. The EG had slightly better results on tests of functional performance, but this was not significant.

There are limitations of this study. It is possible that the intervention was too short to reveal the effects of EMG-BF. Patients were admitted to inpatient rehabilitation rather late in their recovery and there were significant variations in the postoperative day among the sample, with a relatively broad range between the earliest and the latest postoperative day of enrolment. The use of EMG-BF in earlier rehabilitation phases might reveal better effectiveness of the device. Only one final assessment was performed, after the intervention, without follow up, and the potential long-term effects remain unknown. Only functional tests were used to assess the m. quadriceps femoris strength and function. Torque and voluntary activation of the m. quadriceps femoris measurement would improve the quality of the trial. Also, differences between the EG and CG in the proportion of patients operated on in general hospitals using the lateral surgical approach within the subgroup of early starters, and differences in the two domains of SF-36 within the subgroup of late starters could have influenced the results, so we consider them another limitation of the study.

It would be useful to examine the effects of EMG-BF in earlier phases of the rehabilitation, as well as long-term effects in future trials. Also, it would be interesting to compare different combinations of isometric and dynamic exercise with the supplementation of the EMG-BF. Furthermore, it would be useful to study the effect of biofeedback-assisted exercise on hip abductor strength, because it is decreased after THA.

In conclusion, our results suggest no additional benefit from the supplementation of the EMG-BF to the conventional physiotherapy protocol consisting of exercise, electrotherapy, and education after THA. Conventional rehabilitation in both groups improved quality of life and motor function. These results should be confirmed by future studies.

## Figures and Tables

**Table 1:** Baseline characteristics for the experimental and control groups.

Variable	EG (N = 41)	CG (N = 42)	*p*
Age (years; mean (SD))	63.9 (8.8)	63.9 (9)	0.999 ^a^
Body height (cm; mean (SD))	170.5 (9.8)	172 (8.1)	0.444 ^a^
Body mass (kg; mean (SD))	83.9 (15)	85.9 (16.4)	0.577 ^a^
BMI (kg/m^2^; mean (SD))	28.8 (4.5)	28.8 (4.2)	0.966 ^a^
Sex (N(%))			
Male	25 (61)	25 (59.5)	1.000 ^b^
Female	16 (39)	17 (40.5)	
Education (N(%))			
Primary level	1 (2.4)	2 (4.8)	0.563 ^c^
Secondary level	35 (85.4)	32 (76.2)	
Tertiary level	5 (12.2)	8 (19)	
Side of the operated hip (N(%))			
Left	23 (56.1)	19 (45.2)	0.383 ^b^
Right	18 (43.9)	23 (54.8)	
Surgical approach			
Anterior	13 (13.7)	10 (23.8)	0.406 ^c^
Lateral	27 (65.9)	32 (76.2)	
Posterior	1 (2.4)	0 (0)	
Place of the surgery (N(%))			
University hospital	15 (36.6)	14 (33.3)	0.820 ^b^
General hospital	26 (63.4)	28 (66.7)	
Postoperative day (day; median [IQR])	89 [75–125.5]	90.5 [61.5–114.8]	0.645 ^d^
Use of mobility aid (N(%))			
Unilateral crutch	26 (63.4)	17 (40.5)	0.167 ^c^
Two crutches	9 (22)	13 (31)	
Walker	0 (0)	1 (2.4)	
No walking aid	6 (14.6)	11 (26.2)	
HOOS score (0–100 scale; mean (SD) or median [IQR])			
Pain	83 [70–95]	78 [68–90.8]	0.364 ^d^
Symptoms	80 [67.5–87.5]	80 [70–85]	0.856 ^d^
ADL function	70.5 (19.4)	70.3 (15.4)	0.953 ^a^
Sport and recreation function	54.1 (22.8)	50.6 (19.8)	0.459 ^a^
Quality of life	42.4 (25.4)	40.3 (22.2)	0.682 ^a^
SF-36 domains (0–100 scale; mean (SD) or median [IQR])			
Physical functioning	43 (19.6)	43.1 (21.5)	0.992 ^a^
Role physical	0 [0–25]	0 [0–31.3]	0.172 ^d^
Role emotional	0 [0–50]	0 [0–33.3]	0.794 ^d^
Vitality	56.9 (19.6)	54.2 (17.9)	0.514 ^a^
Mental health	44.9 (12.8)	46.2 (11.3)	0.620 ^a^
Social functioning	58 [41.5–72.8]	56 [41.5–71.6]	0.840 ^d^
Bodily pain	41.5 [12.5–70.5]	41.5 [25–70.5]	0.779 ^d^
General health	65 [55–77.5]	62.5 [45–75]	0.176 ^d^
PCS	45.1 (6.5)	45.1 (6.9)	0.977 ^a^
MCS	45 (6)	44.6 (6.1)	0.752 ^a^
NRS (0–10 scale; median [IQR])	2 [0–2]	2 [0–3.3]	0.162 ^d^
30 s CST (no. of stands; median [IQR])	11 [8.5–13]	10 [7–12]	0.103 ^d^
TUG (seconds; median [IQR])	12.5 [10.9–15]	13 [9.6–15.9]	0.792 ^d^

EG—experimental group; CG—control group; SD—standard deviation; IQR—interquartile range; N—sample size; BMI—body mass index; HOOS—Hip Disability and Osteoarthritis Outcome Score; ADL—activities of daily living; SF-36—Short Form Health Survey-36; PCS—physical component summary score; MCS—mental component summary score; NRS—Numeric Rating Scale; 30 s CST—thirty-second chair stand test; TUG—Timed Up and Go test; ^a^ *t*-test for independent samples; ^b^ Fisher’s exact test; ^c^ chi-square test; ^d^ Mann–Whitney U test.

**Table 2:** Baseline characteristics of the participants regarding the start of the rehabilitation.

Variable	Early Start (N = 43)			Late Start (N = 40)		
	EG (N = 21)	CG (N = 22)	*p*	EG (N = 20)	CG (N = 20)	*p*
Age (years; mean (SD))	63.8 (9.1)	61.5 (8.3)	0.390 ^a^	64.1 (8.8)	66.6 (9.1)	0.373 ^a^
Body height (cm; mean (SD))	171 (10.5)	173 (7)	0.444 ^a^	170.1 (9.2)	171 (9.2)	0.771 ^a^
Body mass (kg; mean (SD))	85.8 (15.3)	87.7 (17.8)	0.707 ^a^	82 (14.7)	83.8 (14.9)	0.695 ^a^
BMI (kg/m^2^; mean (SD))	29.3 (4.4)	29.1 (4.6)	0.898 ^a^	28.3 (4.6)	28.5 (3.8)	0.849 ^a^
Sex (N(%))						
Male	8 (38.1)	7 (31.8)	0.755 ^b^	8 (40)	10 (50)	0.751 ^b^
Female	13 (61.9)	15 (68.2)		12 (60)	10 (50)	
Education (N(%))						
Primary level	0 (0)	0 (0)	0.698 ^c^	1 (5)	2 (10)	0.149 ^c^
Secondary level	17 (81)	19 (86.4)		18 (90)	13 (65)	
Tertiary level	4 (57.1)	3 (13.6)		1 (5)	5 (25)	
Side of the operated hip (N(%))						
Left	10 (47.6)	9 (40.9)	0.763 ^b^	13 (65)	10 (50)	0.523 ^b^
Right	11 (52.4)	13 (59.1)		7 (35)	10 (50)	
Surgical approach						
Anterior	9 (42.9)	3 (13.6)	0.047 ^c,^*	4 (20)	7 (35)	0.480 ^b^
Lateral	11 (52.4)	19 (86.4)		16 (80)	13 (65)	
Posterior	1 (4.8)	0 (0)		0 (0)	0 (0)	
Place of the surgery (N(%))						
University hospital	10 (47.6)	3 (13.6)	0.022 ^b,^*	5 (25)	11 (55)	0.105 ^b^
General hospital	11 (52.4)	19 (86.4)		15 (75)	9 (45)	
Postoperative day (day; median [IQR])	77 [49–79]	63 [48–82.3]	0.601 ^d^	125.5 [106.3–140.5]	115.5 [103–143.5]	0.620 ^d^
Use of mobility aid (N(%))						
Unilateral crutch	13 (61.9)	9 (40.9)	0.370 ^c^	13 (65)	8 (40)	0.285 ^c^
Two crutches	5 (23.8)	9 (40.9)		4 (20)	4 (20)	
Walker	0 (0)	0 (0)		0 (0)	1 (5)	
No walking aid	3 (14.3)	4 (18.2)		3 (15)	7 (35)	
HOOS score (0–100 scale; mean (SD) or median [IQR])						
Pain	83 [64–96.5]	76.5 [66.8–95]	0.670 ^d^	83 [73–95]	78 [69.3–88]	0.429 ^d^
Symptoms	75 [67.5–90]	77.5 [63.8–85]	0.581 ^d^	80 [66.3–85]	80 [75–85]	0.779 ^d^
ADL function	68.3 (17.5)	67 (14.7)	0.788 ^a^	72.8 (21.3)	73.9 (15.7)	0.854 ^a^
Sport and recreation function	51 (25.6)	49.9 (15.2)	0.861 ^a^	57.3 (19.6)	51.4 (24.2)	0.402 ^a^
Quality of life	37.2 (21.4)	38.2 (20.8)	0.884 ^a^	47.9 (28.6)	42.6 (24)	0.529 ^a^
SF-36 domains (0–100 scale; mean (SD) or median [IQR])						
Physical functioning	39.5 (17.4)	40.9 (22.7)	0.824 ^a^	57.9 (21.4)	49.1 (17.4)	0.147 ^a^
Role physical	0 [0–0]	0 [0–25]	0.189 ^d^	0 [0–87.5]	0 [0–31.3]	0.201 ^d^
Role emotional	0 [0–83.3]	16.7 [0–33.3]	0.829 ^d^	66.7 [16.7–100]	0 [0–100]	0.041 ^d,^*
Vitality	56.5 (21.1)	54 (20.7)	0.689 ^a^	70.8 (12.2)	65.6 (19.6)	0.306 ^a^
Mental health	45.2 (14.3)	47.5 (11)	0.563 ^a^	40 [32.5–57.5]	42.5 [30–60]	0.845 ^d^
Social functioning	54 [31–58]	56 [41.5–71.6]	0.455 ^d^	87.5 [58–100]	64.3 [41.5–87.5]	0.080 ^d^
Bodily pain	41.5 [12.5–70.5]	41.5 [22.9–70.5]	0.633 ^d^	66.5 [47.8–91.5]	58 [28–73.6]	0.470 ^d^
General health	65 [60–80]	65 [45–75]	0.134 ^d^	75 [65–85]	65 [58.8–75]	0.044 ^d,^*
PCS	43.9 (5.4)	44 (6.7)	0.978 ^a^	51 (8.7)	47.1 (7.1)	0.111 ^a^
MCS	45 (6.7)	44.9 (6.7)	0.960 ^a^	48.7 (5.2)	45.7 (8.1)	0.159 ^a^
NRS (0–10 scale; median [IQR])	2 [0–2]	2 [0–3]	0.255 ^d^	1.5 [0–2.8]	2 [0–4.8]	0.383 ^d^
30 s CST (no. of stands; median [IQR])	10 [9–13]	10 [7–12]	0.186 ^d^	11 [7.3–13]	9 [7–11.8]	0.314 ^d^
TUG (seconds; median [IQR])	12.5 [10.9–13.3]	13.5 [10.3–15.9]	0.319 ^d^	12.3 [10.8–15.3]	12.5 [8.7–18.1]	0.640 ^d^

EG—experimental group; CG—control group; SD—standard deviation; IQR—interquartile range; N—sample size; BMI—body mass index; HOOS—Hip Disability and Osteoarthritis Outcome Score; ADL—activities of daily living; SF-36—Short Form Health Survey-36; PCS—physical component summary score; MCS—mental component summary score; NRS—Numeric Rating Scale; 30 s CST—thirty-second chair stand test; TUG—Timed Up and Go test; ^a^ *t*-test for independent samples; ^b^ Fisher’s exact test; ^c^ chi-square test; ^d^ Mann–Whitney U test; * statistically significant.

**Table 3:** SF-36 results before and after the intervention.

SF-36 Domains	Pre-Intervention Mean (SD) or Median [IQR]	Post-Intervention Mean (SD) or Median [IQR]	*p*
EG (N = 41)			
Physical functioning	43 (19.6)	57.8 (22)	<0.001 ^a,^*
Role physical	0 [0–25]	0 [0–50]	0.001 ^b,^*
Role emotional	0 [0–50]	66.7 [0–100]	0.003 ^b,^*
Vitality	56.9 (19.6)	65.4 (14.9)	0.009 ^a,^*
Mental health	44.9 (12.8)	41.1 (14.6)	0.268 ^a^
Social functioning	58 [41.5–72.8]	70.5 [49.8–100]	0.004 ^b,^*
Bodily pain	41.5 [12.5–70.5]	58 [37.5–85.3]	0.002 ^b,^*
General health	65 [55–77.5]	70 [60–85]	0.046 ^b,^*
PCS	45.1 (6.5)	50.4 (8.4)	<0.001 ^a,^*
MCS	45 (6)	46.8 (6.1)	0.177 ^a^
CG (N = 42)			
Physical functioning	43.1 (21.5)	51.9 (18.5)	0.002 ^a,^*
Role physical	0 [0–31.3]	0 [0–31.3]	0.712 ^b^
Role emotional	0 [0–33.3]	0 [0–100]	0.177 ^b^
Vitality	54.2 (17.9)	62.4 (19.4)	0.015 ^a,^*
Mental health	45 [40–55]	45 [30–60]	0.470 ^b^
Social functioning	56 [41.5–71.6]	70.5 [41.5–84.1]	0.020 ^b,^*
Bodily pain	41.5 [25–70.5]	58 [28–70.5]	0.030 ^b,^*
General health	62.5 [45–75]	65 [55–80]	0.038 ^b,^*
PCS	45.1 (6.9)	48.1 [44.1–54.5]	<0.001 ^a,^*
MCS	44.6 (6.1)	45.5 [38–53.1]	0.519 ^a^

EG—experimental group; CG—control group; SD—standard deviation; IQR—interquartile range; N—sample size; SF-36—Short Form Health Survey-36; PCS—physical component summary score; MCS—mental component summary score; ^a^ *t*-test for paired samples; ^b^ Wilcoxon signed-rank test; * statistically significant.

**Table 4:** Mobility aid use, HOOS, SF-36, NRS, 30 s CST, and TUG results post-intervention.

Variable	EG (N = 41)	CG (N = 42)	*p*
Use of mobility aid (N(%))			
Unilateral crutch	12 (29.3)	11 (26.2)	0.789 ^a^
Two crutches	3 (7.3)	3 (7.1)	
Walker	0 (0)	1 (2.4)	
No walking aid	26 (63.4)	27 (64.3)	
HOOS score (0–100 scale; mean (SD) or median [IQR])			
Pain	93 [83–98]	89 [75–93.5]	0.121 ^b^
Symptoms	85 [80–95]	85 [75–95]	0.267 ^b^
ADL function	87 [75.5–93]	81.5 [70.5–93]	0.452 ^b^
Sport and recreation function	63 [56–88]	66 [48.5–76.5]	0.522 ^b^
Quality of life	54.5 (25.5)	51.3 (23.7)	0.555 ^c^
SF-36 domains (0–100 scale; mean (SD) or median [IQR])			
Physical functioning	57.8 (22)	51.9 (18.5)	0.190 ^c^
Role physical	0 [0–50]	0 [0–31.3]	0.389 ^b^
Role emotional	66.7 [0–100]	0 [0–100]	0.051 ^b^
Vitality	65.4 (14.9)	62.4 (19.4)	0.427 ^c^
Mental health	40 [30–55]	45 [30–60]	0.497 ^b^
Social functioning	70.5 [49.8–100]	70.5 [41.5–84.1]	0.183 ^b^
Bodily pain	58 [37.5–85.3]	58 [28–70.5]	0.444 ^b^
General health	70 [60–85]	65 [55–80]	0.157 ^b^
PCS	50.4 (8.4)	48 (6.8)	0.161 ^c^
MCS	46.8 (6.1)	45.4 (7.7)	0.365 ^c^
NRS (0–10 scale; median [IQR])	0 [0–2]	0.5 [0–2]	0.237 ^b^
30 s CST (no. of stands; median [IQR])	14 [11–17]	12.5 [10–15.3]	0.076 ^b^
TUG (seconds; median [IQR])	8.7 [6.9–11.5]	9.2 [7.3–11.7]	0.716 ^b^

EG—experimental group; CG—control group; SD—standard deviation; IQR—interquartile range; N—sample size; HOOS—Hip Disability and Osteoarthritis Outcome Score; ADL—activities of daily living; SF-36—Short Form Health Survey-36; PCS—physical component summary score; MCS—mental component summary score; NRS—Numeric Rating Scale; 30 s CST—thirty-second chair stand test; TUG—Timed Up and Go test. ^a^ chi-square test; ^b^ Mann–Whitney U test; ^c^ *t*-test for independent samples.

## Data Availability

The data presented in this study are available on request from the corresponding author.
